# Statin use and breast cancer-specific mortality and recurrence: a systematic review and meta-analysis including the role of immortal time bias and tumour characteristics

**DOI:** 10.1038/s41416-025-03070-w

**Published:** 2025-06-12

**Authors:** Oliver William Scott, Sandar Tin Tin, Alana Cavadino, J. Mark Elwood

**Affiliations:** 1 Department of Oncology, School of Medical Sciences, University of Auckland, Auckland, New Zealand; 2 Department of Epidemiology and Biostatistics, School of Population Health, University of Auckland, Auckland, New Zealand

**Keywords:** Breast cancer, Cancer epidemiology

## Abstract

**Background:**

The association between statins and breast cancer-specific mortality and recurrence has been examined in several previous observational studies and meta-analyses. However, potentially important effect modifiers have not often been explored in previous meta-analyses. In this study, an updated systematic review and meta-analysis was undertaken to ascertain the association between statins and both breast cancer death (BCD) and breast cancer recurrence (BCR).

**Methods:**

Articles were sourced from various databases up until the 13th of June 2024, and effect estimates were pooled using the random effects model. Subgroup analyses were conducted by the potential for immortal time bias (ITB), type of statin (lipophilic vs hydrophilic), estrogen receptor status (positive vs negative), stage (‘early’ vs ‘advanced’), and type of postdiagnostic use (‘new’ vs ‘prevalent’ user).

**Results:**

Pooled results showed that there was a statistically significant protective association between statin use and both BCD (21 studies, hazard ratio = 0.81, 95% CI: 0.75–0.87) and BCR (20 studies, HR = 0.81, 95% CI: 0.74–0.89). Lipophilic statins were more protective than hydrophilic statins with BCD as the outcome, and there were suggestions of a more protective association in studies with ITB and in ER+ patients with BCR as the outcome. There was little evidence of effect modification by stage or type of postdiagnostic use.

**Conclusion:**

In this meta-analysis, we observed that statin use, particularly lipophilic statin use, was associated with favourable outcomes for BCD and BCR.

## Background

Breast cancer is the most common cancer in women and the leading cause of female cancer mortality worldwide [1]. Comorbidities are common in patients with breast cancer [2], and there is a high and increasing prevalence of risk factors for both breast cancer and ischaemic heart disease among Western women [3–5]. As such, many patients with breast cancer use prescribed medications for cardiovascular conditions. Ascertaining the association between commonly used cardiovascular medications and breast cancer-specific mortality and recurrence is therefore warranted. Statins (3-hydroxy-3-methylglutaryl coenzyme A reductase inhibitors) are the most widely prescribed cholesterol lowering medications [6] and are used for both the primary and secondary prevention of cardiovascular disease [7].

Statins reduce cholesterol levels by inhibiting the rate limiting enzyme of the mevalonate pathway (HMGCR), which has been shown to be over expressed in breast cancer tumours [8, 9]. Statins have been found to exert pleiotropic effects, such as the induction of apoptosis, inhibition of proliferation, as well as expressing immunomodulatory properties [10–12]. In preclinical studies, statins have also reported to be associated with anti-neoplastic properties in animal models and breast cancer cell lines [13–15]. On the basis of this evidence, a number of observational studies have been carried out, and several meta-analyses have been published to summarise these studies [16–24]. Published between 2015 and 2023 and covering studies published up until the 1^st^ of June 2023, most of these meta-analyses have shown a statistically significant protective association between the use of statins and the prognosis of breast cancer.

In the meta-analyses published to date, some of the more recent observational studies have not been included [25–29]. Some of the meta-analyses have also missed previously published observational studies, perhaps owing to insufficient and/or incomplete literature search strategies or different eligibility criteria being used. Further, there have not been any meta-analyses that have examined potentially important effect modifiers of the association such as the presence of immortal time bias (ITB), the stage of breast cancer (‘early’ vs ‘advanced’ stage), or the type of postdiagnostic statin use (‘new’ vs ‘prevalent’ user). ITB is a bias in which a spurious survival advantage is generally conferred to the user group in studies that count user time in a period where events could not occur by design [30]. For example, if a woman is dispensed a statin at a certain point in time after her diagnosis, it is not possible that she died between her diagnosis and the statin dispensing. Moreover, recent experimental and observational studies have indicated that statins may exert their effect on late-stage tumour progression/metastasis [13, 31–33], and knowing if the effect of statins is modified by stage would provide important information to clinicians when prescribing statins to breast cancer patients. Finally, it is widely acknowledged in the pharmacoepidemiological literature that drug initiators (i.e., ‘new’ users) will likely differ on important health characteristics relative to existing (or ‘prevalent’) users [34]. While the type of statin (lipophilic vs hydrophilic) and patients’ estrogen receptor (ER) status (positive vs negative) have been examined in subgroup analyses in some previous meta-analyses, we also wanted to assess these variables as potential effect modifiers in updated subgroup analyses. As such, the objective of the current paper was to perform an updated systematic review and meta-analysis examining the association between statins and both breast cancer death (BCD) and breast cancer recurrence (BCR), and also examine whether the associations differ across potentially important subgroups.

## Methods

### Literature search/data sources

PubMed, Embase, Medline, Web of Science, Google Scholar, and the Cochrane Library databases were searched using the keywords [statin, hydroxymethylglutaryl-CoA reductase inhibitor, HMG-CoA reductase inhibitor, cholesterol lowering, simvastatin, atorvastatin, pravastatin, lovastatin, fluvastatin, pitavastatin, breast cancer, breast carcinoma, cancer, carcinoma, recurrence, metastasis, outcome, death, prognosis, survival, mortality, proliferation] in combinations of ‘AND’ or ‘OR’. There was no restriction placed on the earliest date of publication, but articles were only selected for review if they analysed human breast cancer patients and were written in English. Further articles were sourced from scanning the reference lists of individual observational studies, systematic reviews, and meta-analyses found using the aforementioned search criteria. Literature was sourced up until the 13th of June 2024.

### Study selection/eligibility criteria

Studies were eligible for inclusion if they met the following criteria: (1) Included women with breast cancer as the cohort of interest; (2) Analysed data from cohort studies, case-control studies, or randomised controlled trials (including retrospective analyses of already published RCTs); (3) Assessed statin use as the exposure of interest (with no minimum dose requirement); (4) Reported BCD or BCR (or both) as outcomes of interest; (5) Reported 95% confidence intervals or standard errors for the associations of interest; and (6) Were published as full length articles. We did not consider laboratory experiments, case reports, ecological studies, conference abstracts, or editorials for inclusion in this meta-analysis.

### Data extraction

The titles and abstracts of each individual study were first examined to assess their eligibility, and relevant information from each study was then independently extracted by two authors (OS and STT). We subsequently liaised with each other to resolve any disagreements and come to a consensus. Information extracted included the first author and year of study, country of study, the number of women under study, study design (prospective cohort vs retrospective cohort; prospective cohort studies were those that actively enroled a cohort of patients and followed them up over time, while retrospective cohort studies were studies that retrospectively analysed already recorded/collected data), patient characteristics (such as estrogen receptor status, cancer stage, and age), exposure definition (pre or postdiagnosis), type of postdiagnostic use (‘new’ vs ‘prevalent’ user; ‘new’ postdiagnostic users were defined as statin users who did not have a prescription/dispensing in a specified time period prior to diagnosis, while ‘prevalent’ postdiagnostic users were defined as statin users who could have also used statins prior to diagnosis), the source of exposure data, median or mean follow up time, the potential for ITB (studies were judged to be susceptible to ITB when they used a ‘time fixed’ postdiagnosis definition of medication use; these studies erroneously counted the time between a woman’s cancer diagnosis and first statin prescription/dispensing as user time instead of correctly classifying it as nonuser time), covariates adjusted for, the types of statins used by women (e.g., all statins, lipophilic statins, hydrophilic statins, or a combination), and the reference group used (e.g. statin users vs all statin nonusers or statin users vs a different comparison group). When studies used a stepwise approach for covariate adjustment, the fully adjusted HR was taken.

### Statistical analysis

The main analysis assessed the association between statin use at any time and breast cancer-specific mortality and recurrence, pooling the data from all included studies. When studies reported risk estimates for statins used both before and after breast cancer diagnosis for the same cohort, only the risk estimates for postdiagnosis use were taken (to avoid sample overlap), as it can be considered that postdiagnosis use of medications over the course of breast cancer therapy is the more clinically relevant exposure period. Risk estimates and 95% confidence intervals were transformed onto the log scale, as recommended [35]. To pool risk estimates into a summary estimate, the inverse variance method with random effects model was used. We used a random effects model because it seems plausible that the effect of statins could vary from study to study due to factors other than sampling variability [36]. In the paper by Li and others [37], the HRs for the associations between statin use and BCR in all patients and statin use and BCR in ER+ and ER- patients were initially stratified by length of statin use (<3 years, 3–5 years, and 5+ years), and no overall HR was reported. These HRs were first pooled using a random-effects model to derive an overall summary estimate for any statin use, and the resulting pooled HRs were then included in this meta-analysis. A similar method was used to pool results for the associations between statin use and BCR in all patients and statin use and BCR in ER- patients in the paper by Sim and colleagues [38]. The same method was again employed to derive overall summary estimates for lipophilic statin use in the papers by Murtola et al. and Nowakowska et al. [39, 40] and in ‘early’ stage (nonmetastatic) patients in the papers by Lofling and colleagues and Murto et al. [25, 26]. Finally, the same method was again used to pool results in ER+ and ER- patients for both BCD and BCR in the paper by Guo and others [29] and to derive an overall summary estimate for BCR in the paper by Dumas and colleagues [27]. The Higgins I^2^ statistic was computed for each pooled estimate to ascertain the amount of heterogeneity present between studies, and an I^2^ statistic of >50% indicated that there was a significant amount of heterogeneity [41].

To explore reasons for heterogeneity between studies and to assess the impact of different potential modifying variables on the associations of interest, subgroup analyses [42] were carried out by the potential for ITB (yes vs no), the type of statin used (lipophilic vs hydrophilic), ER status (positive vs negative), the stage of breast cancer (‘early’ vs ‘advanced’), and the type of postdiagnostic use (‘new’ vs ‘prevalent’ user). The intention of the subgroup analysis by stage was to analyse metastatic (‘advanced’ stage) patients vs all others, however due to the relative dearth of studies that stratified their analyses by stage, any study that did so was included in this subgroup analysis instead of studies that exclusively examined metastatic patients vs others. All studies examining BCR as an outcome of interest in this meta-analysis only included ‘early’ stage (nonmetastatic) patients, and this subgroup analysis was therefore restricted to BCD only. Because of the bias inherent in studies judged to be susceptible to ITB [30], the subgroup analyses by type of statin, ER status, stage of breast cancer, and type of postdiagnostic use were restricted to studies judged not to be susceptible to ITB. Furthermore, because studies that assess medication use prior to cancer diagnosis are inherently protected from ITB, we carried out an additional subgroup analysis by ITB in studies that exclusively assessed medication use after cancer diagnosis. To formally test for differences between subgroups, a random effects meta-regression was used [43]. Lastly, funnel plots [44] were generated, and Egger’s regression tests [45] were performed to assess the presence of any potential publication bias. If publication bias was judged to be present, trim and fill analysis [46] was conducted to assess any potential change(s) in the summary estimate(s). All reported *p* values are two-sided and were considered statistically significant if *p* < 0.05. All analyses were conducted in STATA 17.0 (StataCorp, College Station, TX).

## Results

### Literature search

**Fig. 1:**
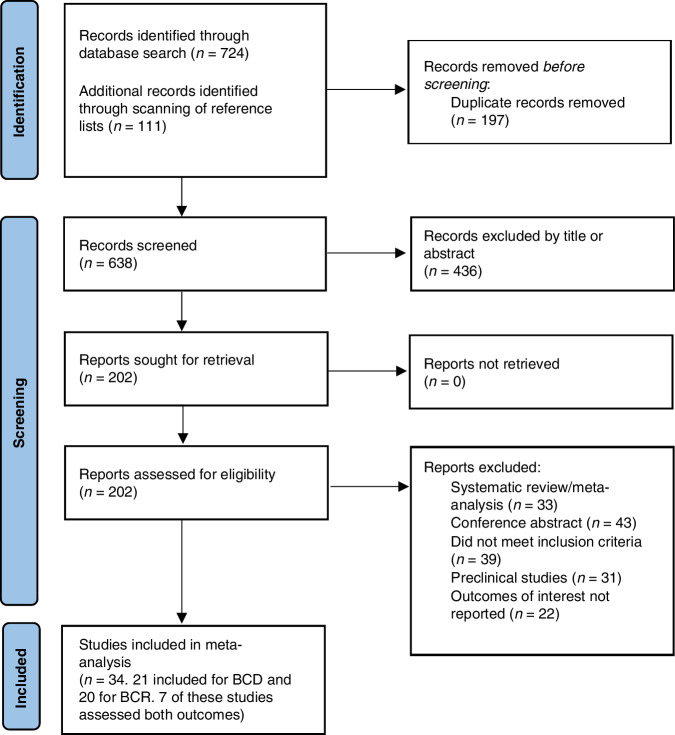
PRISMA chart of study selection.

A total of 638 studies (after removing duplicates) were retrieved from the selected databases during the literature search and included for initial screening (Fig. 1). Two hundred and two reports were assessed in full, and after making various exclusions, 34 studies were deemed to meet the inclusion criteria and were included in this meta-analysis [25–29, 31, 37–40, 47–70]. The detailed characteristics of these 34 studies are shown in Table 1.

**Table 1:** Characteristics of the included studies.

First author and year of study	Country of study	Number under study	Study design	Patient characteristics	Stratification by estrogen receptor status	Stratification by stage	Exposure definition	Type of use (if the exposure period was postdiagnostic)	Source of exposure data	Follow up time (mean or median, in years)	Potential for immortal time bias	Variables adjusted for	Types of statins used by women in study	Reference group used	Study period (first date of BC dx until end of follow up where stated, otherwise dx dates of BC patients included)	Outcome(s) reported (relevant to this meta-analysis)	Effect estimates
Guo et al. [29]	USA	38,858	Retrospective cohort	Women with stage I-III BC. Mean age not reported	Yes	No	Statin use after diagnosis of BC	New users	Prescription records	3.7	No	Age, race, year of diagnosis, stage, grade, cancer surgery, chemotherapy, radiotherapy, hormone therapy, depression, cardiovascular disease, hyperlipidemia, hypertension, and diabetes	All types of statins, hydrophilic and lipophilic in sensitivity analysis	All non-users of statins	2008-2018	BCD and BCR	0.85 (0.75-0.96) and 1.05 (0.91-1.21) for BCD and BCR respectively
Giorello et al. [28]	Argentina	261	Retrospective cohort	Women with stage I-II BC. Mean age not reported	No	No	Statin use after BC surgery	Prevalent users	Medical records	Not reported	Yes	ER status, PR status, tumour size, grade, and type of statin	All types of statins	All non-users of statins	2007-2023	BCR	0.22 (0.08-0.61)
Dumas et al. [27]	France	235,368	Retrospective cohort	Women with stage I-III BC. Median age of 60.0 years	No	No	Statin use in the 6-month period prior to BC diagnosis	-	Prescription records	4.5	No	Age, comorbid conditions, deprivation index, number of GP visits, number of gynaecologist visits, screening performance, number of medications, exposure to other medications, cancer subtype, nodal status, chemotherapy, and endocrine therapy	Lipophilic (simvastatin) and hydrophilic (rosuvastatin) analysed separately	All non-users of statins	2011-2018	BCR	0.73 (0.66-0.82)
Murto et al. [26]	Finland	13,378	Retrospective cohort	Women with all stages of BC. Median age of 62.0 years	Yes	Yes (metastatic/stage 4 patients vs all others)	Statin use both before (from 1995 until BC diagnosis) and after diagnosis of BC	Prevalent users	Prescription records	4.5	No	Age, number of screening rounds, tumour extent, histological characteristics, primary treatment, coronary artery disease, diabetes, hypertension, Charlson comorbidity index score, hormone receptor status, hormone therapy, and total cholesterol	All types of statins	All non-users of statins	1995-2015	BCD	1.22 (1.02-1.46) and 0.85 (0.73-1.00) for pre and postdiagnostic use respectively
Lofling et al. [25]	Norway	26,190	Retrospective cohort	Women with all stages of BC. Median ages of 65.0 and 62.0 years for statin users and nonusers, respectively	Yes	Yes (metastatic/stage 4 patients vs all others)	Statin use both before (in the 3-month period prior) and after diagnosis of BC	Prevalent users	Prescription records	6.1	No	Age, education, marital status, number of children, country of origin, stage, molecular subtype, histology, and post-diagnostic use of concomitant medications (low-dose aspirin, non-aspirin antiplatelets, metformin, non-metformin antidiabetics, NSAIDs, beta blockers, ACEIs, ARBs, calcium channel blockers, and diuretics)	All types of statins	All non-users of statins	2004-2018	BCD	0.90 (0.81-1.01) and 0.84 (0.75-0.94) for pre and postdiagnostic use respectively
Scott et al. [69]	New Zealand	14,976	Retrospective cohort	Women with all stages of BC. Mean age of 58.3 years	Yes	Yes (metastatic/stage 4 patients vs all others)	Statin use both before (in the 1-year period prior to diagnosis) and after diagnosis of BC	Prevalent users	Prescription records	4.5	No	Age, date of diagnosis, ethnic group, deprivation, urban/rural status, public/private status, register, stage, grade, mode of detection, lymphovascular invasion, receptor status, beta blocker use, ACEI use, ARB use, diuretic use, metformin use, tamoxifen use, aromatase inhibitor use, cardiac conditions, diabetes, stroke, COPD, peripheral vascular disease, and renal disease	All types of statins	All non-users of statins, different comparison group in sensitivity analysis	2007-2017	BCD and BCR	0.72 (0.61-0.85) and 0.86 (0.72-1.02) for BCD and BCR respectively (prediagnostic) 0.74 (0.63-0.86) and 0.94 (0.80-1.11) for BCD and BCR respectively (postdiagnostic)
Takada et al. [70]	Japan	719	Retrospective cohort	Women with cT1 BC only. Median age of 58.0 years	No	Only ‘early’ stage patients included	Statin use after diagnosis of BC	Prevalent users	Self-report	5.0	No	Tumour size and hyperlipidemia	All types of statins, hydrophilic and lipophilic in sensitivity analysis	All non-users of statins	2007-2020	BCR	0.35 (0.05-7.15)
Inasu et al. [31]	Sweden	360	Prospective cohort	Postmenopausal women with all stages of BC. Median age of 68.0 years for statin users	No	No	Statin use after diagnosis of BC	New users	Prescription records	8.6	No	Age, year of dx, grade, BMI, ER status, nodal status, tumour size, surgery, chemotherapy, radiotherapy, and endocrine therapy	All types of statins	All non-users of statins	2005-2020	BCR	0.88 (0.82-0.96)
Sim et al. [38]	Singapore	7858	Retrospective cohort	Women with stage II-III BC. Mean age not reported	Yes	Only ‘early’ stage patients included	Statin use after diagnosis of BC	New users	Prescription records	8.8	No	Age, stage, grade, ER status, HER2 status, aspirin use, diabetes, hypertension, CVD, neurological disease, renal disease, and hyperlipidemia	All types of statins	All non-users of statins	2005-2015	BCD and BCR	1.05 (0.83-1.33) and 0.72 (0.36-1.41) for BCD and BCR respectively
Nowakowska et al. [40]	USA	23,192	Retrospective cohort	Women with stage I-III BC. Mean age not reported	Yes	Only ‘early’ stage patients included	Statin use in the 1-year period after diagnosis of BC	New users	Prescription records	3.3	No	Age, race, year of dx, education, SES, residential area, stage, tumour size, number of lymph nodes involved, molecular subtype, surgery, chemotherapy, radiotherapy, endocrine therapy, baseline hypertension, and Charlson comorbidity index score	All types of statins, hydrophilic and lipophilic in sensitivity analysis	All non-users of statins	2008-2015	BCD	0.83 (0.66-1.04)
Bjarnadottir et al. [68]	Sweden	910	Prospective cohort	Women with stage I-III BC. Mean age of 65.5 years	No	Only ‘early’ stage patients included	Statin use both before and after diagnosis of BC (in the same analysis)	-	Prescription records	Not reported	Yes	Age, grade, tumour size, LVI, ER status, and adjuvant treatment	All types of statins	All non-users of statins	1991-2016	BCD	0.80 (0.34-1.91)
Harborg et al. [67]	Denmark	14,773	Retrospective cohort	Postmenopausal women with stage I-III BC. Median age of 65.0 years	No	Only ‘early’ stage patients included	Statin use after diagnosis of BC	New users	Prescription records	4.5	No	Age, stage, grade, type of surgery, chemotherapy, radiotherapy, metformin use, aspirin use, HRT use, and Charlson comorbidity index score	All types of statins, lipophilic and specific types of statins in sensitivity analysis	All non-users of statins	2007-2018	BCR	0.72 (0.50-1.04)
Hosio et al. [66]	Finland	3533	Retrospective cohort	Women with all stages of BC. Median age of 72.0 years	No	No	Statin use in the 3-year period prior to BC diagnosis	-	Prescription records	4.6	No	Age, calendar year, stage, metformin use, insulin use, other oral anti-diabetic use, and duration of type 2 diabetes	All types of statins	All non-users of statins	1998-2013	BCD	0.76 (0.63-0.92)
Borgquist et al. [65]	Sweden	20,559	Retrospective cohort	Women with all stages of BC. Mean age of 64.0 years	No	No	Statin use both before (requiring at least two prescriptions) and after diagnosis of BC	Prevalent users	Prescription records	5.1	No	Age, stage, and diabetes	All types of statins, hydrophilic and lipophilic in sensitivity analysis	All non-users of statins	2005-2012	BCD	0.77 (0.63-0.95) and 0.83 (0.75-0.93) for pre and postdiagnostic use respectively
Li et al. [37]	USA	1523	Retrospective cohort	Women with stage II-III BC. Mean age of 64.9 years	Yes	Only ‘early’ stage patients included	Statin use both before and after diagnosis of BC (in the same analysis)	-	Medical records	5.9	Yes	Age, tumour size, stage, grade, extracapsular extension, tumour subtype, and Charlson comorbidity index score	All types of statins	All non-users of statins	1995-2015	BCR	0.54 (0.17-1.69)
Tryggvadottir et al. [64]	Sweden	985	Prospective cohort	Women with stage I-III BC. Mean age of 61.0 years	No	Only ‘early’ stage patients included	Statin use after diagnosis of BC	Prevalent users	Self-report	7.0	Yes	Age, BMI, tumour size, lymph node involvement, grade, ER status, and alcohol use	All types of statins	All non-users of statins	2002-2016	BCR	0.64 (0.23-1.78)
Haukka et al. [63]	Finland	4,288	Retrospective cohort	Women with all stages of BC. Mean age not reported	No	No	Statin use after diagnosis of BC	Prevalent users	Prescription records	2.0	No	Age, diabetes, stage, and calendar period	All types of statins	All non-users of statins	1996-2012	BCD	0.71 (0.59-0.86)
Shaitelman et al. [62]	USA	869	Retrospective cohort	Women with stage I-III BC (TNBC only). Median age of 51.0 years	Only ER- patients included	Only ‘early’ stage patients included	Statin use after diagnosis of BC	Prevalent users	Medical records	6.3	Yes	Age, BMI, stage, grade, and cancer treatment	All types of statins	All non-users of statins	1997-2012	BCD and BCR	0.70 (0.47-1.03) and 0.82 (0.57-1.16) for BCD and BCR respectively
Smith et al. [61]	Ireland	6314	Retrospective cohort	Women with stage I-III BC. Mean age of 68.1 years	Yes	Only ‘early’ stage patients included	Statin use prior to BC diagnosis (time period not specified)	-	Prescription records	Not reported	No	Age, smoking status, stage, grade, molecular subtype, chemotherapy, LVI, screen detected cancer, tumour size, anti-ER therapy, aspirin use, anti-diabetic use, NSAID use, bisphosphonate use, and comorbidity score	All types of statins, hydrophilic and lipophilic in sensitivity analysis	All non-users of statins	2001-2011	BCD	0.81 (0.68-0.96)
McMenamin et al. [60]	Scotland	15,140	Retrospective cohort	Women with all stages of BC. Mean age not reported	Yes	No	Statin use both before (in the 1-year period prior) and after diagnosis of BC	Prevalent users	Prescription records	4.0	No	Age, year of dx, SES, stage, grade, molecular subtype, cancer treatment, aspirin use, HRT use, metformin use, hormone therapy use, myocardial infarction, CHF, peripheral vascular disease, stroke, COPD, peptic ulcer, liver disease, diabetes, and renal disease	All types of statins, hydrophilic, lipophilic, and specific types of statins in sensitivity analysis	All non-users of statins	2009-2015	BCD	0.85 (0.74-0.98) and 0.95 (0.79-1.15) for pre and postdiagnostic use respectively
Sakellakis et al. [59]	Greece	610	Retrospective cohort	Women with stage I-III BC. Mean age of 56.8 years	Sensitivity analysis carried out in ER+ patients only	Only ‘early’ stage patients included	Statin use after diagnosis of BC	Prevalent users	Medical records	3.4	No	Age, stage, hormone receptor status, and HER2 status	All types of statins	All non-users of statins	1983-2013	BCR	0.39 (0.11-1.4)
Smith et al. [58]	Ireland	4243	Retrospective cohort	Women with stage I-III BC. Median ages of 66.0 and 65.0 years for nonusers and users of statins respectively	No	Only ‘early’ stage patients included	Statin use after diagnosis of BC	New users	Prescription records	4.9	No	Age, smoking status, stage, grade, molecular subtype, chemotherapy, anti-ER therapy, aspirin use, anti-diabetic use, NSAID use, bisphosphonate use, and comorbidity score	All types of statins, hydrophilic and lipophilic in sensitivity analysis	All non-users of statins	2001-2012	BCD	0.88 (0.66-1.17)
Cardwell et al. [57]	England	17,880	Retrospective cohort	Women with all stages of BC. Mean age not reported	No	Yes (stage 1 and 2 patients vs stage 3 and 4 patients)	Statin use both before (in the 1-year period prior) and after diagnosis of BC	Prevalent users	Prescription records	5.7	No	Age, smoking status, alcohol, SES, year of dx, stage, grade, surgery, chemotherapy, radiotherapy, BMI, anti-ER use, aspirin use, beta blocker use, metformin use, stroke, COPD, CHF, diabetes, myocardial infarction, peptic ulcer, peripheral vascular disease, and renal disease	All types of statins, hydrophilic and lipophilic in sensitivity analysis	All non-users of statins, active comparator of glaucoma medication users in sensitivity analysis	1998-2012	BCD	0.81 (0.71-0.93) and 0.84 (0.68-1.04) for pre and postdiagnostic use respectively
Desai et al. [56]	USA	128,675	Retrospective cohort	Postmenopausal women with all stages of BC. Mean age of 63.4 years	No	No	Statin use both at baseline (of the WHI trial) and after diagnosis of BC	Prevalent users	Self-report	11.5	No	Age at menarche, smoking, race, education, number of births, BMI, waist circumference, screening, gail 5-year risk, female relative with BC, breast biopsy, hysterectomy, hormone use, OC use, and aspirin use	All types of statins, hydrophilic and lipophilic in sensitivity analysis	All non-users of statins	1998-2010	BCD	0.91 (0.60-1.37) and 0.59 (0.32-1.06) for baseline and time-varying statin use respectively
Boudreau et al. [55]	USA	4216	Retrospective cohort	Women with stage I-II BC. Median age of 63.0 years	No	Only ‘early’ stage patients included	Statin use after diagnosis of BC	Prevalent users	Prescription records	6.3	No	Age, year of dx, stage, hormone receptor status, cancer treatment, endocrine therapy, BMI, smoking status, menopausal status, Charlson comorbidity index score, diabetes, NSAID use, aspirin use, screening, and concurrent medication use (ACEIs, BBs, CCBs, and diuretics)	All types of statins, hydrophilic and lipophilic in sensitivity analysis	All non-users of statins	1990-2011	BCR	0.89 (0.74-1.08)
Lacerda et al. [54]	USA	519	Retrospective cohort	Women with stage I-III BC. Mean age of 61.0 years	No	Only ‘early’ stage patients included	Statin use after diagnosis of BC	Prevalent users	Medical records	2.5	No	Age, BMI, stage, grade, menopausal status, race, nodal status, ER and PR status, HER2 status, lymphovascular invasion, and hormonal therapy	All types of statins	All non-users of statins	1995-2011	BCR	0.40 (0.16-1.00)
Murtola et al. [39]	Finland	31,236	Retrospective cohort	Women with all stages of BC. Median age of 58.6 years	Yes	Yes (metastatic/stage 4 patients vs all others)	Statin use both before (in the 1-year period prior) and after diagnosis of BC	Prevalent users	Prescription records	3.3	No	Age, stage, morphology, and cancer treatment	All types of statins, hydrophilic and lipophilic in sensitivity analysis	All non-users of cholesterol lowering drugs	1995-2003	BCD	0.54 (0.44-0.67) and 0.46 (0.38-0.55) for pre and postdiagnostic use respectively
Botteri et al. [53]	Italy	800	Retrospective cohort	Postmenopausal women with stage I-III BC (TNBC only). Mean age not reported	Only ER- patients included	Only ‘early’ stage patients included	Statin use at the time of breast cancer diagnosis (no timeframe used)	-	Medical records	5.7	No	Age, stage, cancer treatment, and concurrent medication use	All types of statins	All non-users of statins	1997-2008	BCD and BCR	0.89 (0.43-1.85) and 1.11 (0.66-1.88) for BCD and BCR respectively
Brewer et al. [52]	USA	723	Retrospective cohort	Women with stage III BC (inflammatory only). Mean age of 49.6 years	No	Only ‘early’ stage patients included	Statin use after diagnosis of BC	Prevalent users	Medical records	2.9	No	Age, race, stage, grade, BMI, menopausal status, LVI, chemotherapy, radiotherapy, hormonal therapy, surgery, ACEI use, ARB use, BB use, bisphosphonate use, insulin use, metformin use, diabetes, and hypertension	All types of statins, hydrophilic and lipophilic in sensitivity analysis	All non-users of statins	1995-2011	BCD and BCR	0.95 (0.58-1.56) and 0.63 (0.42-0.96) for BCD and BCR respectively
Nickels et al. [51]	Germany	3189	Retrospective cohort	Women with all stages of BC (for BCD). Stage four patients were excluded when switching the outcome to BCR. Mean age not reported	No	No	Current exposure vs never/past exposure (after diagnosis of BC)	New users	Self-report	5.3 and 5.4 years for BCD and BCR respectively	Yes	Age, smoking status, tumour size, nodal status, metastases, BMI, mode of detection, radiotherapy, HRT use, CVD, and diabetes	All types of statins	All non-users (and past users) of statins	2001-2009	BCD and BCR	1.04 (0.67-1.60) and 0.83 (0.54-1.24) for BCD and BCR respectively
Nielsen et al. [50]	Denmark	46,562	Retrospective cohort	Women with all stages of BC. Mean age not reported	No	No	Statin use in the 2-year period prior to BC diagnosis	-	Prescription records	3.6	No	Age, birth year, descent, education, residential area, stage, chemotherapy, radiotherapy, CVD, and diabetes	All types of statins	All non-users of statins	1995-2009	BCD	0.88 (0.80-0.99)
Ahern et al. [49]	Denmark	18,769	Prospective cohort	Women with stage I-III BC. Mean age not reported	Yes	Only ‘early’ stage patients included	Statin use after diagnosis of BC	Prevalent users	Prescription records	6.8	No	Age, menopausal status, grade, ER status, adjuvant therapy, type of surgery, HRT use, aspirin use, ACEI use, NSAID use, and anticoagulant use	All types of statins, lipophilic, hydrophilic, and specific types of statins in sensitivity analysis	All non-users of statins, different comparison group (hydrophilic statins) when analysing simvastatin	1996-2008	BCR	0.83 (0.70-0.98)
Chae et al. [48]	USA	703	Retrospective cohort	Women with stage II-III BC. Mean age of 59.1 years	No	Only ‘early’ stage patients included	Statin use after diagnosis of BC	Prevalent users	Medical records	4.6	Yes	Age, race, menopausal status, family history of BC, smoking status, diabetes, hormone receptor status, hormonal therapy, ACEI use, and ARB use	All types of statins	All non-users of statins	1999-2008	BCR	0.48 (0.28-0.82)
Kwan et al. [47]	USA	1811	Prospective cohort	Women with stage I-IIIa BC. Mean age of 58.4 years	No	Only ‘early’ stage patients included	Statin use after diagnosis of BC	New users	Prescription records	5.0	No	Age, race, stage, BMI, and tamoxifen use	All types of statins	All non-users of statins	1997-2006	BCR	0.67 (0.39-1.13)

### Study characteristics

Overall, this meta-analysis included 34 studies [25–29, 31, 37–40, 47–70] with a combined total of 689,990 women with breast cancer. Twenty-one of these studies assessed BCD as an outcome, while twenty assessed BCR as an outcome (seven of these studies assessed both outcomes). Twenty of the 34 studies were conducted in Europe (including the UK), ten in North America, three from the Asia/Oceania region, and one was from South America. Twenty-nine were designed as retrospective cohort studies and five were prospective cohort studies. All studies adjusted for covariates, and every study (except for two [28, 70]) adjusted for age at the very least. In general, a range of confounders were usually adjusted for, including demographic variables, breast cancer clinical variables, comorbidities, and concomitant medication usage. For example, potentially important confounders such as cancer stage, comorbidities (or an index of the severity of comorbid conditions), and concomitant medication use (excluding hormonal therapy) were adjusted for in 25/34, 20/34, and 16/34 studies, respectively. The mean or median follow up time across the 34 studies ranged from 2.0 to 11.5 years, with sixteen studies reporting a mean/median follow up time of 0–5 years, fourteen reporting a mean/median follow up time of 5–10 years, and one reporting a mean/median follow up time of 10+ years. Follow-up time was not reported in three studies. Twenty-seven studies analysed statin use after the diagnosis of breast cancer (of these, eight included ‘new’ users only, while 19 analysed ‘prevalent’ users), five considered prediagnosis statin use as the exposure of interest, and two analysed both periods concurrently. Twenty-two studies ascertained medication use through prescription records, eight through medical records, and four through self-reported patient data. Twenty-seven studies were judged not to be susceptible to ITB, while seven were. There were fourteen studies that stratified statin use by type (lipophilic vs hydrophilic), one that analysed only lipophilic statins in a sensitivity analysis, while nineteen did not stratify by the type of statin. Eleven studies stratified cancers by their ER status (positive vs negative), two included only ER- patients, one analysed only ER+ patients in a sensitivity analysis, while twenty did not stratify by ER status. Finally, there were five studies that stratified by cancer stage (‘early’ vs ‘advanced’), twenty-one that included only ‘early’ stage (nonmetastatic) patients, while eight did not stratify by cancer stage.

### Association between statin use and breast cancer related deaths

**Fig. 2:**
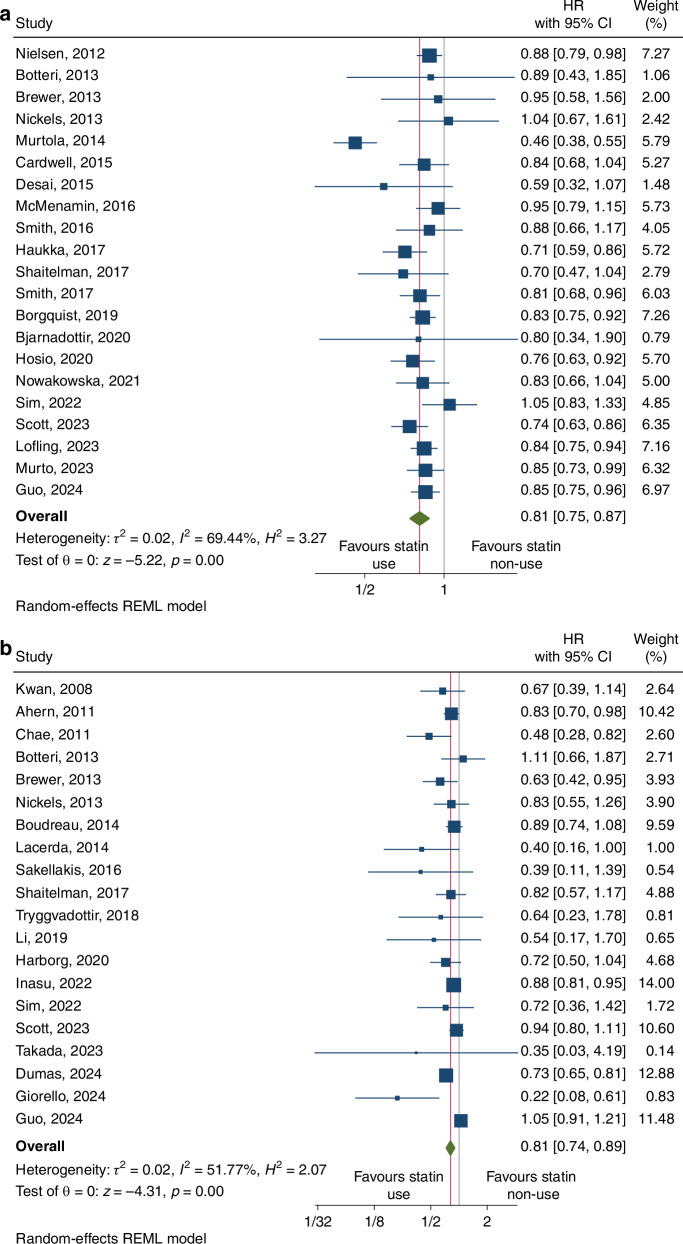
Forest plots for studies assessing the association between statin use and breast cancer prognosis. Legends (**a**: breast cancer specific mortality; **b** breast cancer recurrence).

Overall, 21 studies were included in the meta-analysis examining the association between statin use and breast cancer-specific mortality. Pooled results with a random effects model showed that there was a statistically significant protective association between statin use and BCD (HR = 0.81, 95% CI: 0.75–0.87, *p* < 0.001; Fig. 2a). There was significant heterogeneity between these studies (*I*^2^ = 69.44%).

Subgroup analyses showed that the type of statin used may affect the outcome. In the studies that examined lipophilic statin use, the results indicated that these statins were associated with a statistically significant protective association on BCD (HR = 0.75, 95% CI: 0.62–0.91, *p* = 0.003, Table 2 and Supplementary Fig. 1), and the association with hydrophilic statins also indicated a statistically significant protective association, but smaller in magnitude (HR = 0.93, 95% CI: 0.89–0.97, *p* = 0.001; p for subgroup difference = 0.05). Other analyses revealed that stratifying the association by other potential modifiers (presence of ITB, estrogen receptor status, stage, and type of postdiagnostic use) did not significantly change the results (*p* values for subgroup differences all > 0.05; Table 2, Supplementary Fig. 1 and Supplementary Table 1). Furthermore, in studies that exclusively assessed medication use after cancer diagnosis, there was no difference found when stratifying the association by the presence of ITB.

**Table 2:** Subgroup meta-analyses.

Study characteristics	Breast cancer specific mortality					Breast cancer recurrence				
	Number per subgroup	HR (95% CI)	*I*^2^ (%)	*P*^a^	*P*^b^	Number per subgroup	HR (95% CI)	*I*^2^ (%)	*P*^a^	*P*^b^
Immortal time bias^c^										
No	18 [25, 26, 29, 38–40, 50, 52, 53, 56–58, 60, 61, 63, 65, 66, 69]	0.81 (0.74–0.88)	73	**<0.001**		14 [27, 29, 31, 38, 47, 49, 52–55, 59, 67, 69, 70]	0.84 (0.76–0.93)	54	**<0.001**	
Yes	3 [51, 62, 68]	0.83 (0.61–1.14)	16	0.25	0.83	6 [28, 37, 48, 51, 62, 64]	0.62 (0.45–0.87)	43	**0.006**	0.09
Type of statin^d^										
Hydrophilic	10 [29, 39, 40, 52, 56–58, 60, 61, 65]	0.93 (0.89–0.97)	0	**0.001**		6 [27, 29, 49, 52, 55, 70]	0.83 (0.62–1.10)	68	0.19	
Lipophilic	10 [29, 39, 40, 52, 56–58, 60, 61, 65]	0.75 (0.62–0.91)	88	**0.003**	**0.05**	7 [27, 29, 49, 52, 55, 67, 70]	0.81 (0.68–0.95)	54	**0.01**	0.80
Estrogen receptor status^d^										
Negative	10 [25, 26, 29, 38–40, 53, 60, 61, 69]	0.87 (0.63–1.21)	89	0.41		5 [29, 38, 49, 53, 69]	1.07 (0.88–1.31)	0	0.51	
Positive	9 [25, 26, 29, 38–40, 60, 61, 69]	0.80 (0.73–0.89)	42	**<0.001**	0.53	5 [29, 38, 49, 59, 69]	0.76 (0.59–0.98)	77	**0.03**	0.08
Stage_d_										
‘Early’ stage	12 [25, 26, 29, 38–40, 52, 53, 57, 58, 61, 69]	0.80 (0.68–0.95)	85	**0.009**		-	-	-	-	-
‘Advanced’ stage	5 [25, 26, 39, 57, 69]	0.75 (0.52–1.08)	81	0.13	0.74	-	-	-	-	-

Bold text indicates a statistically significant *p* value.

^a^*P* values for specific subgroup hazard ratio.

^b^*P* values for subgroup differences. The differences were tested through a random-effects meta regression.

^c^For the ‘immortal time bias’ subgroup, ‘yes’ means that individual studies were deemed to be susceptible to immortal time bias, while ‘no’ means they were not.

^d^The ‘type of statin’, ‘estrogen receptor status’, and ‘stage’ subgroup analyses were restricted to studies judged not to be susceptible to ITB.

### Association between statin use and breast cancer recurrence

Overall, 20 studies were included in the meta-analysis examining the association between statin use and breast cancer recurrence. Pooled results with a random effects model showed that there was a statistically significant protective association between statin use and BCR (HR = 0.81, 95% CI: 0.74-0.89, *p* < 0.001; Fig. 2b). There was significant heterogeneity between these studies (*I*^2^ = 51.77%).

Subgroup analyses revealed that stratifying the association by potential modifiers (presence of ITB, type of statin, estrogen receptor status, and type of postdiagnostic use) did not significantly change the results (*p* values for subgroup differences all > 0.05; Table 2, Supplementary Fig. 2, and Supplementary Table 1). The pooled HRs were lower for studies judged to be susceptible to ITB relative to studies judged not to be susceptible to ITB (in all studies as well as in studies that exclusively assessed medication use after cancer diagnosis) and for patients with ER+ cancers relative to patients with ER- cancers, however these differences were not statistically significant.

### Publication bias

**Fig. 3:**
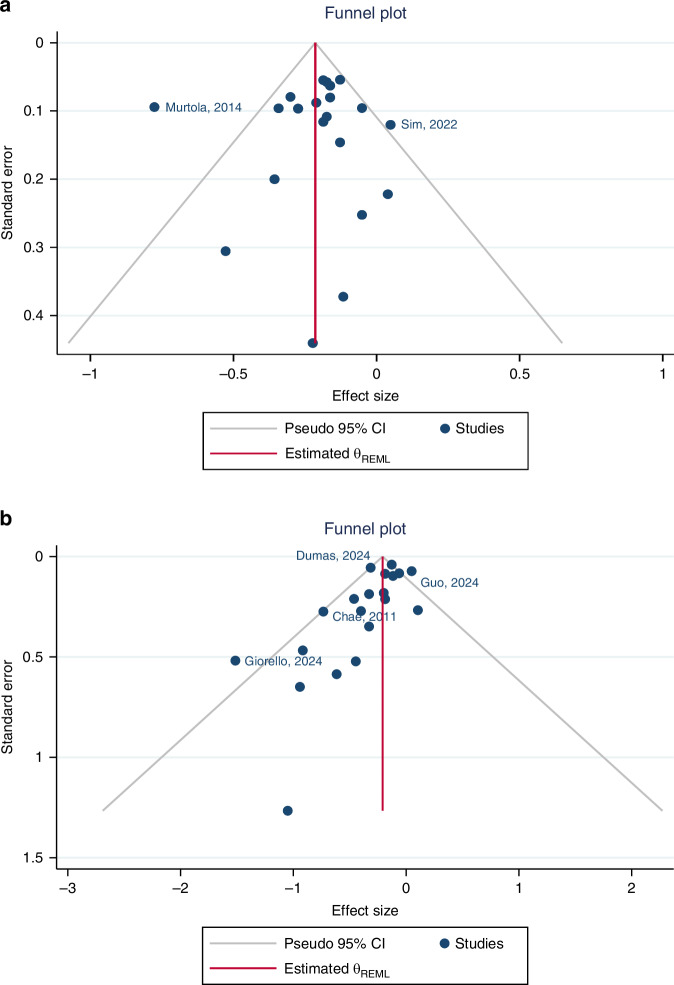
Funnel plots for studies assessing the association between statin use and breast cancer prognosis. The *X* axis represents HRs on the log scale. Legends (**a**: breast cancer specific mortality; **b** breast cancer recurrence).

There was little evidence of publication bias or small study effects (*p* value for small study effects = 0.94) for studies examining the association between statin use and BCD (Fig. 3a). Conversely, the funnel plot for the association between statin use and BCR showed clear evidence of asymmetry (Fig. 3b). In this plot, there was asymmetry in terms of the number of studies on either side of the average effect size, with the plot having more studies on the left-hand side (meaning that there were more studies that showed a lower HR than the overall average effect size relative to studies that showed a higher HR). Further, there was clear evidence of small study effects in this plot, with a big cluster of small studies with large effect sizes toward the bottom left of the plot. The result of Egger’s regression test was in concordance with a visual inspection of the funnel plot (*p* value for small study effects = 0.002). When excluding studies judged to be susceptible to ITB and performing trim and fill analysis [46] on studies assessing BCR as an outcome, there was still a statistically significant protective association shown between statin use and BCR (HR = 0.86, 95% CI: 0.79–0.95; Supplementary Fig. 3).

## Discussion

In this systematic review and meta-analysis, we found a statistically significant protective association between statin use and both BCD and BCR, with a 19% reduction in risk for both outcomes. Our findings are consistent with almost every other meta-analysis examining the association between statin use and breast cancer prognosis [16–24]. Every previous meta-analysis that has examined BCR as an outcome has found a statistically significant protective association with statin use, and all but one previous meta-analysis (which was published in 2016) [18] has found a statistically significant protective association between statin use and BCD. In the 2016 meta-analysis by Manthravadi and colleagues, the association between statin use and BCD was bordering on statistical significance (HR = 0.70, 95% CI: 0.46–1.06, *p* = 0.09).

Immortal time bias (ITB) is a bias introduced in pharmacoepidemiological studies in which a survival advantage is generally conferred to the user group by way of misattributing user time over a period where events could not occur by design [30]. For example, if a postdiagnostic exposure period is used and a binary yes/no indicator is used to model medication use (i.e., a time fixed approach), the time between cancer diagnosis and the first medication dispensing is ‘immortal’ because the patient needed to have survived this period to be dispensed a medication. Lévesque and colleagues showed that medications with no biological basis for doing so (e.g., NSAIDS) could be made to show a decreased risk of diabetes progression by modelling medication use through a time fixed approach [71]. The subgroup analyses by ITB in this meta-analysis did not show the pattern we were expecting, with both outcomes showing no statistically significant difference between studies judged to be susceptible to ITB relative to studies judged not to be susceptible to ITB (for both the ‘main’ ITB analysis as well as the ITB analysis in which we exclusively considered postdiagnostic use). However, the difference between groups with BCR as the outcome was approaching statistical significance in both ITB analyses. To our knowledge, this is the first meta-analysis in this area to examine ITB as a potential effect modifier. When considering BCD as the outcome in the ‘main’ ITB analysis, there were only three studies that were judged to be susceptible to ITB. Further, some studies that were not judged to be susceptible to ITB, such as those by Murtola and colleagues [39], Sakellakis and colleagues [59], and Takada et al. [70] all showed very strong protective associations (i.e., a very low HR) between statin use and BCD/BCR. The HRs derived from these studies could be considered outliers, and there were no obvious reasons to explain the very low HRs derived in these studies.

In the subgroup analysis by type of statin, there was evidence of lipophilic statins being more efficacious than hydrophilic statins with BCD as the outcome, however, there was no effect modification shown with BCR as the outcome. The result with BCD is consistent with preclinical studies, which have indicated that only lipophilic statins have anti-proliferative effects on breast cancer cells [14, 72, 73]. Several previous meta-analyses have also examined the hydrophilicity of statins as a potential effect modifier, with two showing no evidence of effect modification for either outcome [18, 20], one also showing a more protective association for lipophilic statins with BCD as the outcome [19], and a recent meta-analysis by Jaiswal and colleagues showing a more protective association for lipophilic statins with BCR as the outcome [23]. The differing results by type of statin with BCR as the outcome between the meta-analysis by Jaiswal and others and the current meta-analysis can likely be explained by the additional inclusion of the studies by Brewer et al. [52], Dumas and others [27], and Guo et al. [29] in the current meta-analysis, which all showed a slightly lower HR (statistically non-significant differences) for hydrophilic statin users.

There have been hypotheses that statins may be more efficacious in ER+ breast cancers relative to ER- cancers. The more protective effect in ER+ tumours is thought to result from statins lowering levels of cholesterol metabolite 27-hydroxycholesterol (27HC), a selective estrogen receptor modulator that can regulate ER-dependent tumour growth [74–76]. The subgroup analysis by ER status with BCD as the outcome did not show any effect modification; however, the same analysis with BCR as the outcome was bordering on statistical significance, with a strong suggestion that statins were only protective in ER+ cancers. The number of studies available for this subgroup analysis with BCR as the outcome was small, and more studies would be desirable. One previous meta-analysis has also examined ER status as a potential effect modifier, and it found no effect modification for either outcome [21]. However, this meta-analysis erroneously included the study by Desai and colleagues [56] that examined the association between statins and late-stage breast cancer (i.e., incidence instead of prognosis), incorrectly classified the study by Ahern and others [49] as examining BCD instead of BCR, and also included a study by Borgquist et al. that examined all cholesterol lowering medications instead of statins exclusively [77]. The studies by Li et al. [37] and Shaitelman et al. [62] were also excluded from this subgroup analysis in the current meta-analysis due to them being judged to be susceptible to ITB.

Recent experimental data has shown that atorvastatin treatment significantly reduced the metastatic spread of breast cancer to the liver and lung, but had little impact on primary tumour cells [13]. A recent observational study also showed that statins were more efficacious in preventing distant metastasis than locoregional recurrence [31]. These findings suggest that statins may be more protective in advanced-stage patients relative to early-stage patients. To our knowledge, this is the first meta-analysis in this area to examine breast cancer stage as a potential effect modifier, and we were unable to identify any evidence of effect modification. It is worth mentioning that the number of studies in the ‘advanced’ stage arm was small, as well as the fact that this subgroup analysis included the studies by Murtola and others [39] and Murto et al. [26] which both showed a more protective association (statistically significant difference in Murto et al.) in ‘early’ stage patients.

The final subgroup analysis we carried out was by type of postdiagnostic use (‘new’ vs ‘prevalent’ user). We were unable to identify any evidence of effect modification for either outcome in this analysis. To the best of our knowledge, the type of postdiagnostic use has not been examined as a potential effect modifier in any previous meta-analysis. If statins are ever to be used in an adjuvant setting for breast cancer, it is important to ascertain if the protective effect applies to new drug initiators, as it only possible to prospectively prescribe medications to women with a recent diagnosis.

There was a significant amount of between-study heterogeneity present for both outcomes in this meta-analysis, even in many of the individual arms of the subgroup analyses. As shown in Table 1, different studies were often conducted over different time periods and in different countries, inevitably meaning that statins were often used in different clinical contexts. There was also little uniformity regarding the characteristics of women enroled or analysed, with a range of different cancer stages and ages studied. Similarly, the covariates adjusted for varied widely across studies, with some studies making comprehensive adjustments for a range of covariates, while other studies likely lacked access to a similar range of potential confounders. Finally, postdiagnosis statin use may mean use in the first year after diagnosis only or use any time after diagnosis. All of these factors likely play a role in influencing any effect estimate derived, especially when >1 of these factors differ from study to study.

We found clear evidence of publication bias and small study effects with BCR as the outcome, with evidence of asymmetry in the funnel plot and a cluster of small studies with large and protective effect sizes. As such, removing publication bias would attenuate the overall HR towards the null (1.0). However, even after excluding studies judged to be susceptible to ITB and ‘correcting’ for publication bias through the trim and fill method [46], there was still a statistically significant protective association shown between statin use and BCR (Supplementary Fig. 1).

The main strength of this meta-analysis is the large number of observational studies included and the resulting total number of women with breast cancer analysed (689,990). As evidenced by Supplementary Table 2, some of the meta-analyses published to date have missed previously published observational studies, while this review included every study in the table. Furthermore, we studied both BCD and BCR as outcomes, and the consistency in the results between these outcomes gives us confidence in the interpretation of the meta-analysis. We also conducted subgroup analyses by ITB, type of statin used, ER status, and breast cancer stage, which were all subgroups we were interested in a priori with legitimate methodological or clinical justifications for analysing. Finally, although we did not carry out a formal quality assessment of the studies included in this meta-analysis, ITB is a strong indicator of methodological quality for pharmacoepidemiological studies.

This study is not without its limitations. As mentioned above, there was significant between-study heterogeneity for both outcomes, and as a result, it is difficult to determine if the pooled effect estimates derived are driven by statin use exclusively, extraneous factors such as the study period and/or other modifying factors between studies, or a combination of these. For example, some studies were unable to adjust for indications for statin treatment (cardiovascular disease and/or cholesterol levels), and as such, there is likely to be some residual confounding by indication in these studies. Furthermore, even though a strong effort was made to correctly classify studies in terms of their risk of ITB (including emailing authors to clarify their statin exposure definition), it is possible that some studies were incorrectly classified. Moreover, not every study that stratified by stage did so in a uniform manner, and there was one study in the ‘advanced’ stage subgroup that included patients with stage 3 cancer [57]. Even though evidence suggests that statins may exert a more efficacious effect in postmenopausal women relative to premenopausal women [51, 69, 78, 79], we were unable to conduct a subgroup analysis by menopausal status due to how rarely this was reported. Finally, none of the individual studies included in this review were able to ascertain the adherence of statin use among women who were prescribed/dispensed statins. Such non-adherence would likely attenuate any effect estimates derived toward the null [80], however, it is not possible to quantify the extent of this attenuation without knowing the proportion of non-adherence.

In conclusion, we observed an association between statin use and favourable outcomes for BCD and BCR in this systematic review and meta-analysis. We showed that lipophilic statins may be more efficacious than hydrophilic statins with BCD as the outcome, and there were suggestions that studies judged to be susceptible to ITB and ER+ patients had a lower HR than their respective counterparts with BCR as the outcome. There was little evidence of effect modification by the stage of breast cancer or type of postdiagnostic use. However, there was a significant amount of between-study heterogeneity present for both outcomes, and evidence of publication bias with BCR as the outcome. Further research is warranted in patient subgroups defined by estrogen receptor status and stage to ascertain a targeted population of breast cancer patients that may benefit from statin therapy in the adjuvant setting.

## Supplementary information

Supplementary Material
