# Supplementary material for: Statin use and breast cancer-specific mortality and recurrence: a systematic review and meta-analysis including the role of immortal time bias and tumour characteristics
Source: Br J Cancer. 2025 Jun 12;133(4):539–54. doi: 10.1038/s41416-025-03070-w (PMC12356868; doi:10.1038/s41416-025-03070-w)
Supplement: Supplementary file 1 — Supplementary Material [file 41416_2025_3070_MOESM1_ESM.docx]

**A**

**B**

**C**

**D**

**E**

**F**

**Supplementary Figure 1. Forest plots for subgroup meta-analyses assessing the association between statin use and breast cancer specific mortality. Legends (A: Immortal time bias (Table 2); B: Type of statin (Table 2); C: Estrogen receptor status (Table 2); D: Stage (Table 2); E: Immortal time bias in studies that analysed statin use after the diagnosis of breast cancer (Supplementary Table 1); F: Type of postdiagnostic use (Supplementary Table 1)).**

**A**

**B**

**C**

**D**

**E**

**Supplementary Figure 2. Forest plots for subgroup meta-analyses assessing the association between statin use and breast cancer recurrence. Legends (A: Immortal time bias (Table 2); B: Type of statin (Table 2); C: Estrogen receptor status (Table 2); D: Immortal time bias in studies that analysed statin use after the diagnosis of breast cancer (Supplementary Table 1); E: Type of postdiagnostic use (Supplementary Table 1)).**


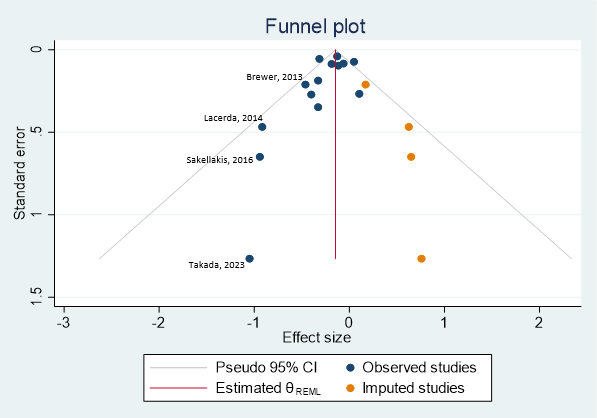


Iteration Number of studies = 18

Model: Random-effects observed = 14

Method: REML imputed = 4

Pooling

Model: Random-effects

Method: REML

---------------------------------------------------------------

Studies | HR [95% conf. interval]

---------------------+-----------------------------------------

Observed | 0.84 0.76 0.93

Observed + Imputed | 0.86 0.79 0.95

---------------------------------------------------------------

**Supplementary Figure 3. Trim and fill analysis for studies assessing the association between statin use and breast cancer recurrence, excluding studies judged to be susceptible to immortal time bias.**

**Supplementary Table 1. Subgroup meta-analyses.**

|  | **Breast cancer specific mortality** | | | | | **Breast cancer recurrence** | | | | |
| --- | --- | --- | --- | --- | --- | --- | --- | --- | --- | --- |
| **Study characteristics** | **Number per subgroup** | **HR (95% CI)** | **I^2^ (%)** | **P_a_** | **P_b_** | **Number per subgroup** | **HR (95% CI)** | **I^2^ (%)** | **P_a_** | **P_b_** |
| **Immortal time bias_c_** |  |  |  |  |  |  |  |  |  |  |
| No | 14 [1-14] | 0.80 (0.72-0.89) | 78 | **<0.001** |  | 12 [1, 10, 11, 14-22] | 0.87 (0.80-0.95) | 31 | **0.002** |  |
| Yes | 2 [23, 24] | 0.84 (0.57-1.24) | 43 | 0.39 | 0.80 | 5 [23-27] | 0.62 (0.43-0.90) | 53 | **0.01** | 0.07 |
| **Type of postdiagnostic use_de_** |  |  |  |  |  |  |  |  |  |  |
| Prevalent users | 10 [1-5, 7, 8, 11-13] | 0.77 (0.67-0.88) | 83 | **<0.001** |  | 7 [1, 11, 16-19, 22] | 0.86 (0.78-0.94) | 0 | **0.001** |  |
| New users | 4 [6, 9, 10, 14] | 0.88 (0.80-0.96) | 0 | **0.006** | 0.19 | 5 [10, 14, 15, 20, 21] | 0.88 (0.75-1.03) | 58 | 0.08 | 0.48 |

*^a^P-values for specific subgroup hazard ratio.*

*^b^P-values for subgroup differences. The differences were tested through a random-effects meta regression.*

*^c^The ‘immortal time bias’ subgroup was restricted to studies that analysed statin use after the diagnosis of breast cancer. ‘Yes’ means that individual studies were deemed to be susceptible to immortal time bias, while ‘no’ means they were not.*

*^d^The ‘type of postdiagnostic use’ subgroup analysis was restricted to studies judged not to be susceptible to ITB.*

*^e^‘New’ postdiagnostic users were defined as statin users who did not have a prescription/dispensing in a specified time period prior to diagnosis, while ‘prevalent’ postdiagnostic users were defined as statin users who could have also used statins prior to diagnosis.*

*^f^****Bold text*** *indicates a statistically significant p-value.*

**Supplementary Table 2. Individual studies included in previous meta-analyses examining the association between statin use and BCD/BCR.**

| Individual study |  | Meta-analyses | | | | | | | |
| --- | --- | --- | --- | --- | --- | --- | --- | --- | --- |
|  | Jia (2023) [28] | Jaiswal (2023) [29] | Zhao (2022) [30] | Xu (2021) [31] | Lv (2020) [32] | Liu (2017) [33] | Manthravadi (2016) [34] | Mansourian (2016) [35] | Wu (2015) [36] |
| Kwan (2008) [15] | ✔ | ✔ | ✔ |  | ✔ |  | ✔ | ✔ |  |
| Ahern (2011) [16] | ✔ | ✔ | ✔ | ✔ | ✔ |  | ✔ | ✔ |  |
| Chae (2011) [25] | ✔ | ✔ | ✔ |  | ✔ |  | ✔ | ✔ |  |
| Nielsen (2012) [37] |  |  |  |  | ✔ |  |  | ✔ |  |
| Botteri (2013) [38] | ✔ |  | ✔ | ✔ | ✔ |  | ✔ |  |  |
| Brewer (2013) [1] | ✔ | ✔ | ✔ |  | ✔ |  | ✔ | ✔ | ✔ |
| Nickels (2013) [23] |  | ✔ |  |  |  | ✔ | ✔ | ✔ | ✔ |
| Boudreau (2014) [17] | ✔ | ✔ | ✔ |  | ✔ |  | ✔ | ✔ |  |
| Lacerda (2014) [18] | ✔ |  | ✔ |  |  |  |  |  |  |
| Murtola (2014) [2] | ✔ | ✔ | ✔ | ✔ | ✔ | ✔ | ✔ | ✔ | ✔ |
| Cardwell (2015) [3] | ✔ | ✔ | ✔ |  | ✔ | ✔ | ✔ |  | ✔ |
| Desai (2015) [4] | ✔ |  |  | ✔ |  | ✔ | ✔ |  | ✔ |
| McMenamin (2016) [5] | ✔ | ✔ | ✔ |  | ✔ | ✔ |  |  |  |
| Sakellakis (2016) [19] | ✔ |  | ✔ |  | ✔ |  |  |  |  |
| Smith (2016) [6] | ✔ |  | ✔ |  |  |  |  |  |  |
| Haukka (2017) [7] | ✔ |  | ✔ |  |  |  |  |  |  |
| Shaitelman (2017) [24] | ✔ |  | * | ✔ | ✔ |  |  |  |  |
| Smith (2017) [39] | ✔ |  | ✔ |  | ✔ |  |  |  |  |
| Tryggvadottir (2018) [26] |  |  | ✔ |  | ✔ |  |  |  |  |
| Borgquist (2019) [8] | ✔ | ✔ | ✔ |  | ✔ |  |  |  |  |
| Li (2019) [40] | ✔ |  | ✔ | ✔ | ✔ |  |  |  |  |
| Bjarnadottir (2020) [41] | ✔ |  | ✔ |  | ✔ |  |  |  |  |
| Harborg (2020) [20] | ✔ | ✔ | ✔ |  |  |  |  |  |  |
| Hosio (2020) [42] | ✔ |  | ✔ |  |  |  |  |  |  |
| Nowakowska (2021) [9] | ✔ |  |  |  |  |  |  |  |  |
| Inasu (2022) [21] | ✔ | ✔ |  |  |  |  |  |  |  |
| Sim (2022) [43] | ✔ | ✔ |  |  |  |  |  |  |  |
| Lofling (2023) [12] |  |  |  |  |  |  |  |  |  |
| Scott (2023) [11] | ✔ | ✔ |  |  |  |  |  |  |  |
| Takada (2023) [22] | ✔ |  |  |  |  |  |  |  |  |
| Murto (2023) [13] |  |  |  |  |  |  |  |  |  |
| Dumas (2024) [44] |  |  |  |  |  |  |  |  |  |
| Giorello (2024) [27] |  |  |  |  |  |  |  |  |  |
| Guo (2024) [14] |  |  |  |  |  |  |  |  |  |

*Note) A tick (✔) indicates that the respective individual study was included in each meta-analysis.*

*Note) A star (*) indicates that the respective individual study was included in the meta-analysis, but not for the outcomes of BCD or BCR.*

*Note) This table does not show studies that were included in previous meta-analyses that we did not include in ours, however there are good reasons why we have not included such studies in our review. In the 2016 review by Mansourian and others, there were three studies included that were conference abstracts (Zeichner, Chavez-Mac Gregor, and Ceacareanu) and one that was a letter to the editor (Sendur). Jaiswal (2023) also included the study by Ceacareanu in their review. Moreover, Jia (2023) included a study by Kim (2021) in which the cohort analysed was the general population (as opposed to breast cancer patients), a study by Chang et al (2023) in which the outcome analysed was all cancer death (as opposed to breast cancer death), and a paper by Lu and colleagues (2020) in which the outcome analysed was second primary cancers (as opposed to breast cancer recurrence).*

1. Brewer TM, Masuda H, Liu DD, Shen Y, Liu P, Iwamoto T, et al. Statin use in primary inflammatory breast cancer: a cohort study. Br J Cancer. 2013;109(2):318–24.

2. Murtola TJ, Visvanathan K, Artama M, Vainio H, Pukkala E. Statin use and breast cancer survival: a nationwide cohort study from Finland. PLoS One. 2014;9(10):e110231.

3. Cardwell CR, Hicks BM, Hughes C, Murray LJ. Statin use after diagnosis of breast cancer and survival: a population-based cohort study. Epidemiology. 2015;26(1):68–78.

4. Desai P, Lehman A, Chlebowski RT, Kwan ML, Arun M, Manson JE, et al. Statins and breast cancer stage and mortality in the Women's Health Initiative. Cancer Causes Control. 2015;26(4):529–39.

5. Mc Menamin UC, Murray LJ, Hughes CM, Cardwell CR. Statin use and breast cancer survival: a nationwide cohort study in Scotland. BMC Cancer. 2016;16(1):600.

6. Smith A, Murphy L, Sharp L, O'Connor D, Gallagher WM, Bennett K, et al. De novo post-diagnosis statin use, breast cancer-specific and overall mortality in women with stage I-III breast cancer. Br J Cancer. 2016;115(5):592–8.

7. Haukka J, Niskanen L, Auvinen A. Risk of cause-specific death in individuals with cancer-modifying role diabetes, statins and metformin. Int J Cancer. 2017;141(12):2437–49.

8. Borgquist S, Broberg P, Tojjar J, Olsson H. Statin use and breast cancer survival - a Swedish nationwide study. BMC Cancer. 2019;19(1):54.

9. Nowakowska MK, Lei X, Thompson MT, Shaitelman SF, Wehner MR, Woodward WA, et al. Association of statin use with clinical outcomes in patients with triple-negative breast cancer. Cancer. 2021;127(22):4142–50.

10. Sim Y, Lim C, Phyu N, Tan KTB, Chew LST, Wong CY, et al. The Impact of Statin Use and Breast Cancer Recurrence - A Retrospective Study in Singapore. Front Oncol. 2022;12:835320.

11. Scott OW, Tin Tin S, Harborg S, Kuper-Hommel MJJ, Lawrenson R, Elwood JM. Post-diagnostic statin use and breast cancer-specific mortality: a population-based cohort study. Breast Cancer Res Treat. 2023;199(1):195–206.

12. Löfling LL, Stoer NC, Andreassen BK, Ursin G, Botteri E. Low-dose aspirin, statins, and metformin and survival in patients with breast cancers: a Norwegian population-based cohort study. Breast Cancer Res. 2023;25(1):101.

13. Murto MO, Simolin N, Arponen O, Siltari A, Artama M, Visvanathan K, et al. Statin Use, Cholesterol Level, and Mortality Among Females With Breast Cancer. JAMA Network Open. 2023;6(11):e2343861-e.

14. Guo H, Malone KE, Heckbert SR, Li CI. Statin use and risks of breast cancer recurrence and mortality. cancer. 2024.

15. Kwan ML, Habel LA, Flick ED, Quesenberry CP, Caan B. Post-diagnosis statin use and breast cancer recurrence in a prospective cohort study of early stage breast cancer survivors. Breast Cancer Res Treat. 2008;109(3):573–9.

16. Ahern TP, Pedersen L, Tarp M, Cronin-Fenton DP, Garne JP, Silliman RA, et al. Statin prescriptions and breast cancer recurrence risk: a Danish nationwide prospective cohort study. J Natl Cancer Inst. 2011;103(19):1461–8.

17. Boudreau DM, Yu O, Chubak J, Wirtz HS, Bowles EJ, Fujii M, et al. Comparative safety of cardiovascular medication use and breast cancer outcomes among women with early stage breast cancer. Breast Cancer Res Treat. 2014;144(2):405–16.

18. Lacerda L, Reddy JP, Liu D, Larson R, Li L, Masuda H, et al. Simvastatin radiosensitizes differentiated and stem-like breast cancer cell lines and is associated with improved local control in inflammatory breast cancer patients treated with postmastectomy radiation. Stem Cells Transl Med. 2014;3(7):849–56.

19. Sakellakis M, Akinosoglou K, Kostaki A, Spyropoulou D, Koutras A. Statins and risk of breast cancer recurrence. Breast Cancer (Dove Med Press). 2016;8:199–205.

20. Harborg S, Heide-Jorgensen U, Ahern TP, Ewertz M, Cronin-Fenton D, Borgquist S. Statin use and breast cancer recurrence in postmenopausal women treated with adjuvant aromatase inhibitors: a Danish population-based cohort study. Breast Cancer Res Treat. 2020;183(1):153–60.

21. Inasu M, Feldt M, Jernstrom H, Borgquist S, Harborg S. Statin use and patterns of breast cancer recurrence in the Malmo Diet and Cancer Study. Breast. 2022;61:123–8.

22. Takada K, Kashiwagi S, Iimori N, Kouhashi R, Yabumoto A, Goto W, et al. Impact of oral statin therapy on clinical outcomes in patients with cT1 breast cancer. BMC Cancer. 2023;23(1):224.

23. Nickels S, Vrieling A, Seibold P, Heinz J, Obi N, Flesch-Janys D, et al. Mortality and recurrence risk in relation to the use of lipid-lowering drugs in a prospective breast cancer patient cohort. PLoS One. 2013;8(9):e75088.

24. Shaitelman SF, Stauder MC, Allen P, Reddy S, Lakoski S, Atkinson B, et al. Impact of statin use on outcomes in triple negative breast cancer. J Cancer. 2017;8(11):2026–32.

25. Chae YK, Valsecchi ME, Kim J, Bianchi AL, Khemasuwan D, Desai A, et al. Reduced risk of breast cancer recurrence in patients using ACE inhibitors, ARBs, and/or statins. Cancer Invest. 2011;29(9):585–93.

26. Tryggvadottir H, Huzell L, Gustbee E, Simonsson M, Markkula A, Jirstrom K, et al. Interactions between ABCB1 genotype and preoperative statin use impact clinical outcomes among breast cancer patients. Front Oncol. 2018;8:428.

27. Giorello MB, Marks MP, Osinalde TM, del Rosario Padin M, Wernicke A, Calvo JC, et al. Post-surgery statin use contributes to favorable outcomes in patients with early breast cancer. Cancer Epidemiology. 2024;90:102573.

28. Jia X, Lu Y, Xu Z, Mu Q. Impact of statin use on breast cancer recurrence and mortality before and after diagnosis: a systematic review and meta-analysis. Frontiers in Oncology. 2023;13.

29. Jaiswal V, Agrawal V, Ang SP, Saleeb M, Ishak A, Hameed M, et al. Post-diagnostic statin use and its association with cancer recurrence and mortality in breast cancer patients: a systematic review and meta-analysis. Eur Heart J Cardiovasc Pharmacother. 2023:pvad057.

30. Zhao G, Ji Y, Ye Q, Ye X, Wo G, Chen X, et al. Effect of statins use on risk and prognosis of breast cancer: a meta-analysis. Anticancer Drugs. 2022;33(1):e507–e18.

31. Xu WH, Zhou YH. The relationship between post-diagnostic statin usage and breast cancer prognosis varies by hormone receptor phenotype: a systemic review and meta-analysis. Arch Gynecol Obstet. 2021;304(5):1315–21.

32. Lv H, Shi D, Fei M, Chen Y, Xie F, Wang Z, et al. Association between statin use and prognosis of breast cancer: a meta-analysis of cohort studies. Front Oncol. 2020;10:556243.

33. Liu B, Yi Z, Guan X, Zeng YX, Ma F. The relationship between statins and breast cancer prognosis varies by statin type and exposure time: a meta-analysis. Breast Cancer Res Treat. 2017;164(1):1–11.

34. Manthravadi S, Shrestha A, Madhusudhana S. Impact of statin use on cancer recurrence and mortality in breast cancer: a systematic review and meta-analysis. Int J Cancer. 2016;139(6):1281–8.

35. Mansourian M, Haghjooy-Javanmard S, Eshraghi A, Vaseghi G, Hayatshahi A, Thomas J. Statins use and risk of breast cancer recurrence and death: a systematic review and meta-analysis of observational studies. J Pharm Pharm Sci. 2016;19(1):72–81.

36. Wu QJ, Tu C, Li YY, Zhu J, Qian KQ, Li WJ, et al. Statin use and breast cancer survival and risk: a systematic review and meta-analysis. Oncotarget. 2015;6(40):42988–3004.

37. Nielsen SF, Nordestgaard BG, Bojesen SE. Statin use and reduced cancer-related mortality. N Engl J Med. 2012;367(19):1792–802.

38. Botteri E, Munzone E, Rotmensz N, Cipolla C, De Giorgi V, Santillo B, et al. Therapeutic effect of beta-blockers in triple-negative breast cancer postmenopausal women. Breast Cancer Res Treat. 2013;140(3):567–75.

39. Smith A, Murphy L, Zgaga L, Barron TI, Bennett K. Pre-diagnostic statin use, lymph node status and mortality in women with stages I-III breast cancer. Br J Cancer. 2017;117(4):588–96.

40. Li YR, Ro V, Steel L, Carrigan E, Nguyen J, Williams A, et al. Impact of long-term lipid-lowering therapy on clinical outcomes in breast cancer. Breast Cancer Res Treat. 2019;176(3):669–77.

41. Bjarnadottir O, Feldt M, Inasu M, Bendahl PO, Elebro K, Kimbung S, et al. Statin use, HMGCR expression, and breast cancer survival - The Malmo Diet and Cancer Study. Sci Rep. 2020;10(1):558.

42. Hosio M, Urpilainen E, Hautakoski A, Marttila M, Arffman M, Sund R, et al. Survival after breast cancer in women with type 2 diabetes using antidiabetic medication and statins: a retrospective cohort study. Acta Oncol. 2020;59(9):1110–7.

43. Sim Y, Lim C, Phyu N, Tan KTB, Chew LST, Wong CY, et al. The impact of statin use and breast cancer recurrence - a retrospective study in Singapore. Front Oncol. 2022;12:1-12.

44. Dumas E, Grandal Rejo B, Gougis P, Houzard S, Abécassis J, Jochum F, et al. Concomitant medication, comorbidity and survival in patients with breast cancer. Nature Communications. 2024;15(1):2966.
